# Hydrogel in the Treatment of Traumatic Brain Injury

**DOI:** 10.34133/bmr.0085

**Published:** 2024-09-26

**Authors:** Shanhe Li, Jiajun Xu, Yuqing Qian, Ruiping Zhang

**Affiliations:** ^1^ Institute of Medical Technology, Shanxi Medical University, Taiyuan 030001, China.; ^2^ The Radiology Department of Shanxi Provincial People’ Hospital Affiliated to Shanxi Medical University, Taiyuan 030001, China.

## Abstract

The high prevalence of traumatic brain injury (TBI) poses an important global public health challenge. Current treatment modalities for TBI primarily involve pharmaceutical interventions and surgical procedures; however, the efficacy of these approaches remains limited. In the field of regenerative medicine, hydrogels have garnered significant attention and research efforts. This review provides an overview of the existing landscape and pathological manifestations of TBI, with a specific emphasis on delineating the therapeutic potential of hydrogels incorporated with various bioactive agents for TBI management. Particularly, the review delves into the utilization and efficacy of hydrogels based on extracellular matrix (ECM), stem cell-loaded, drug-loaded, self-assembled peptide structures or conductive in the context of TBI treatment. These applications are shown to yield favorable outcomes such as tissue damage mitigation, anti-inflammatory effects, attenuation of oxidative stress, anti-apoptotic properties, promotion of neurogenesis, and facilitation of angiogenesis. Lastly, a comprehensive analysis of the merits and constraints associated with hydrogel utilization in TBI treatment is presented, aiming to steer and advance future research endeavors in this domain.

## Introduction

Traumatic brain injury (TBI) is defined as an alteration in brain function, or other evidence of brain pathology, caused by an external force [1]. The incidence of TBI is significant, affecting approximately 69 million individuals annually [2]. Common causes of TBI include traffic accidents, falls, violence, and sports-related injuries [3]. Individuals who have sustained a TBI may exhibit symptoms such as impaired memory and attention deficits [4]. Additionally, psychiatric manifestations like anxiety and depression can arise, potentially contributing to a higher risk of developing neurological conditions like epilepsy and Alzheimer’s disease [5]. Given its substantial morbidity, disability, and mortality rates, TBI has emerged as a significant global public health concern [6–8].

Currently, the primary clinical treatments for TBI consist of pharmacologic and surgical interventions. Among pharmacologic treatments, mannitol or hypertonic saline (HTS) are commonly used to reduce intracranial pressure (ICP) by establishing an osmotic gradient at the blood–brain barrier (BBB) [9]. The efficacy of mannitol and HTS in reducing cumulative ICP burden is unproven, and they are associated with some adverse effects. Mannitol may crystallize in renal tubules, leading to renal impairment, while HTS can raise serum sodium levels, potentially resulting in heart failure and renal failure [9]. Pharmacologic venous thromboembolism (pVTE) prevention is primarily achieved with low molecular weight heparins (LMWHs) such as enoxaparin, nadroparin, and dalteparin. Nevertheless, the incidence of clinical venous thromboembolism is low, and the protective mechanism of pharmacologic prophylaxis remains unclear, necessitating further research [10]. Glucocorticoids (GCs) and nonsteroidal anti-inflammatory drugs (NSAIDs) are employed for the treatment of TBI-induced neuroinflammation. Animal experiments have shown favorable pharmacological effects of anti-inflammatory drugs, but there is insufficient clear evidence of their improvement of clinical outcomes in TBI patients. GCs themselves are prone to various adverse effects. Nonselective NSAIDs exert their anti-inflammatory effects through the inhibition of cyclooxygenase (COX) and can cause peptic ulcers due to the inhibition of COX-1 [11–14]. Additionally, early prophylaxis of post-traumatic seizures (PTSs) can be performed with phenytoin or levetiracetam in TBI patients [15]. The use of seizure prophylaxis is limited to the first week after TBI due to lack of evidence for the efficacy of late PTS prophylaxis [16]. Surgical treatment is mainly employed to address refractory elevated ICP resulting from TBI using decompressive craniectomy (DC). DC has been shown to effectively reduce ICP and shorten intensive care unit stays [17]. Nevertheless, studies have indicated that DC does not improve mortality and may result in poor prognosis [18].

In addition to the conventional treatments mentioned above, therapeutic hypothermia (TH) and hyperbaric oxygen therapy (HBOT) are commonly employed in studies of TBI treatment. Theoretically, TH may mitigate ICP, elevate cerebral perfusion pressure, reduce cerebral oxygen consumption, diminish the release of excitatory neurotransmitters and inflammatory mediators, and uphold the integrity of the BBB [19]. However, studies have indicated that TH may result in adverse neurological outcomes and increased mortality [20]. The definition of HBOT encompasses a therapy involving heightened total atmospheric pressure and partial pressure of oxygen surpassing ambient total and oxygen partial pressures [21]. Although the mechanism of action of HBOT remains unclear, research has demonstrated its potential to foster angiogenesis and neurogenesis and suppress neuroinflammation, brain edema, and apoptosis [22]. It is noteworthy that HBOT exhibits efficacy solely in cases of moderate and severe TBI, not in mild TBI [22,23].

Given the persisting dearth of effective treatments for TBI, biomaterials have emerged as subjects of exploration in TBI treatment research. Among these materials, hydrogels exhibit remarkable biocompatibility and biodegradability, rendering them suitable as carriers for stem cells or pharmaceuticals. They can be precisely localized to the injured brain tissue via minimally invasive techniques, circumventing traditional drug delivery routes that are often ineffective due to the inability to achieve therapeutic concentrations across the BBB. This approach has demonstrated significant efficacy in preclinical studies and holds promise for the development of a novel therapeutic modality for TBI in clinical settings [24–27]. This review synthesizes recent advancements concerning the application of various hydrogel types in TBI, elucidates their advantages and disadvantages, and critically examines potential future directions for their development, with the aim of advancing research efforts focused on TBI treatment.

## Pathophysiologic Changes in TBI

**Fig. 1.**
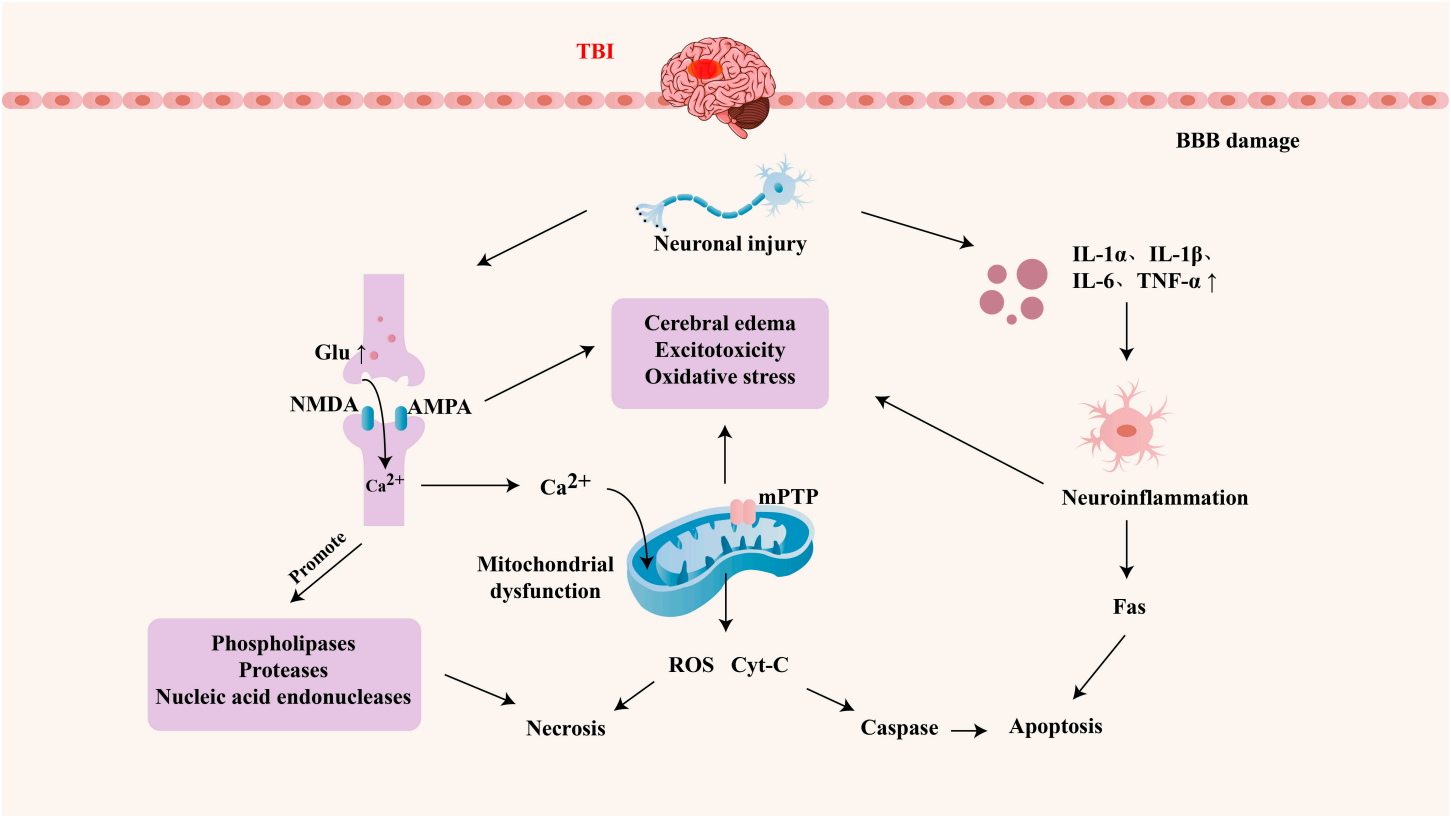
Secondary injury in TBI.

Pathologic changes in TBI are categorized into primary and secondary injuries. The primary injury includes intracranial hemorrhage and cerebral contusion caused by external forces. Secondary injury encompasses a cascade of pathological processes that unfold after the primary injury, primarily involving brain edema, excitotoxicity, oxidative stress, mitochondrial dysfunction, neuroinflammation, and apoptosis (Fig. 1).

### Primary injury

Brain contusion is induced by the deformation of brain tissue and shear force damage resulting from trauma, leading to the demise of neurons, microglia, and astrocytes [28]. Simultaneously, it can trigger vascular rupture, eliciting hemorrhage, ischemia, and other associated symptoms [28]. Trauma-related intracranial hemorrhage encompasses subdural hematoma, epidural hematoma, cerebral hemorrhage, and subarachnoid hemorrhage [29]. Subdural hematoma and epidural hematoma represent common primary injuries after TBI, typically instigating changes such as raised ICP, focal ischemia, and coagulation irregularities [30–32]. Notably, the prevalence and fatality rate of subdural hematoma surpass that of epidural hematoma [33–36]. Subarachnoid hemorrhage arises from bleeding due to cerebral contusion and frequently occurs in TBI cases [37–39]. This type of hemorrhage is linked to an unfavorable prognosis, potentially associated with the cerebral vasospasm and hydrocephalus it induces [37,40,41].

### Secondary injuries

#### Cerebral edema

Cerebral edema is categorized into vasogenic and cytotoxic edema [42]. Trauma-induced cerebral edema primarily manifests as hematogenous cerebral edema [43]. Vasogenic edema results from the initial injury that disrupts the BBB, leading to heightened capillary permeability and extrusion of proteinaceous fluid [43,44]. Edema typically develops at the contusion site and its neighboring region [43].

#### Excitotoxicity

Following TBI, there is an elevated release of excitatory neurotransmitters, such as glutamate, due to mechanical injury and neuronal excitation. High concentrations of glutamate subsequently activate glutamate receptors, which primarily include *N*-methyl-d-aspartic acid (NMDA), α-amino-3-hydroxy-5-methylisoxazole-4-propionate (AMPA), and kainic acid (KA) receptors—3 major ionotropic receptors. The activation of these glutamate receptors leads to an influx of Na^+^ and Ca^2+^ and an efflux of K^+^, resulting in neuronal cell dysfunction and death, a process termed excitotoxicity [45–48]. Increased Ca^2+^ concentration activates calcium-dependent phospholipases, proteases, and nucleic acid endonucleases, which cause protein hydrolysis and DNA damage, ultimately leading to cell death [49]. Concurrently, elevated intracellular Ca^2+^ concentration prompts its entry into the mitochondria, causing a loss of mitochondrial membrane potential. This leads to mitochondrial dysfunction, inhibition of the oxidative phosphorylation process, reduction in ATP synthesis, and an increase in the generation of free radicals, thereby promoting oxidative stress [48,49].

#### Oxidative stress

TBI-induced excitotoxicity, mitochondrial dysfunction, and increased Ca^2+^ concentration can result in elevated production of oxygen free radicals, predominantly reactive oxygen species (ROS), thereby inducing oxidative stress [50,51]. ROS primarily include superoxide ion radical, hydroxyl radical, and hydrogen peroxide, among others. The direct damage to brain tissues at the initial stage of TBI triggers the generation of ROS, followed by excitotoxicity inducing a substantial influx of Ca^2+^, leading to mitochondrial dysfunction and extensive ROS production [49,52]. ROS can cause oxidative damage to DNA, RNA, lipids, and proteins, resulting in cell death. Mitochondrial DNA is particularly susceptible to oxidation and damage by ROS, further promoting mitochondrial dysfunction and the generation of oxygen free radicals that worsen oxidative stress [50]. Furthermore, nitric oxide produced by inducible nitric oxide synthase (iNOS) reacts with superoxide ion radical to produce highly reactive peroxynitrite, a potent oxidizing agent that damages DNA by reacting with guanine [53,54]. Excessive ROS and reactive nitrogen species (RNS) also stimulate the production and release of inflammatory mediators, thus contributing to neuroinflammation [55].

#### Mitochondrial dysfunction

Mitochondrial damage and dysfunction in the mitochondrial oxidative respiratory chain may occur in the early stages of TBI, resulting in disturbances in energy metabolism, insufficient adenosine triphosphate (ATP) synthesis, and the generation of oxidative stress in the form of ROS [56]. TBI-induced tissue damage triggers a significant release of glutamate, activation of NMDA receptors, and influx of large amounts of Ca^2+^, leading to calcium overload, mitochondrial membrane depolarization, ATP synthesis inhibition, dysfunction in the electron transport chain, and impairment of oxidative phosphorylation [57]. Moreover, elevated Ca^2+^ levels activate the mitochondrial permeability transition pore (mPTP), causing an increase in mitochondrial permeability and the release of apoptosis-related proteins such as cytochrome C (Cyt-C); concurrently, cytoplasmic molecules of small to medium size penetrate the mitochondria, resulting in mitochondrial collapse and ultimately culminating in apoptosis and necrosis [58,59].

#### Neuroinflammation

Trauma induces brain tissue damage and the release of intracellular substances from necrotic cells, contributing to inflammation. The mechanical injury resulting from TBI disrupts the BBB and up-regulates the expression of aquaporin channel protein 4 (AQP4) on endothelial cells, facilitating the diffusion of free water through AQP4 channels. This process leads to BBB breakdown, leukocyte recruitment to the injury site, release of inflammatory mediators, and activation of glial cells [60–63]. Following TBI, there is a rapid increase in interleukin-1α (IL-1α), IL-1β, IL-6, and tumor necrosis factor-α (TNF-α) levels, accompanied by ongoing infiltration of inflammatory cells and cytokines into the central nervous system (CNS). This not only directly harms brain tissues but also triggers the activation of brain immune cells, thereby exacerbating the inflammatory responses [64,65]. Besides the injury mechanisms, elevated levels of inflammatory mediators can support nerve regeneration and repair by promoting neural stem cell (NSC) proliferation and differentiation and activating astrocytes and oligodendrocytes [66,67].

#### Apoptosis

TBI precipitates the programmed cell death of neurons and glial cells, a process termed apoptosis. Following trauma, apoptosis manifests through both endogenous and exogenous pathways [68]. The endogenous pathway involves the release of Cyt-C, initiating the caspase cascade predominantly mediated by cysteine aspartases, while the exogenous pathway is activated by the binding of TNF and Fas to their ligands, triggering caspase activation [58,68,69].

## The Therapeutic Mechanism of Hydrogel for TBI

### Extracellular matrix-based hydrogels

**Fig. 2.**
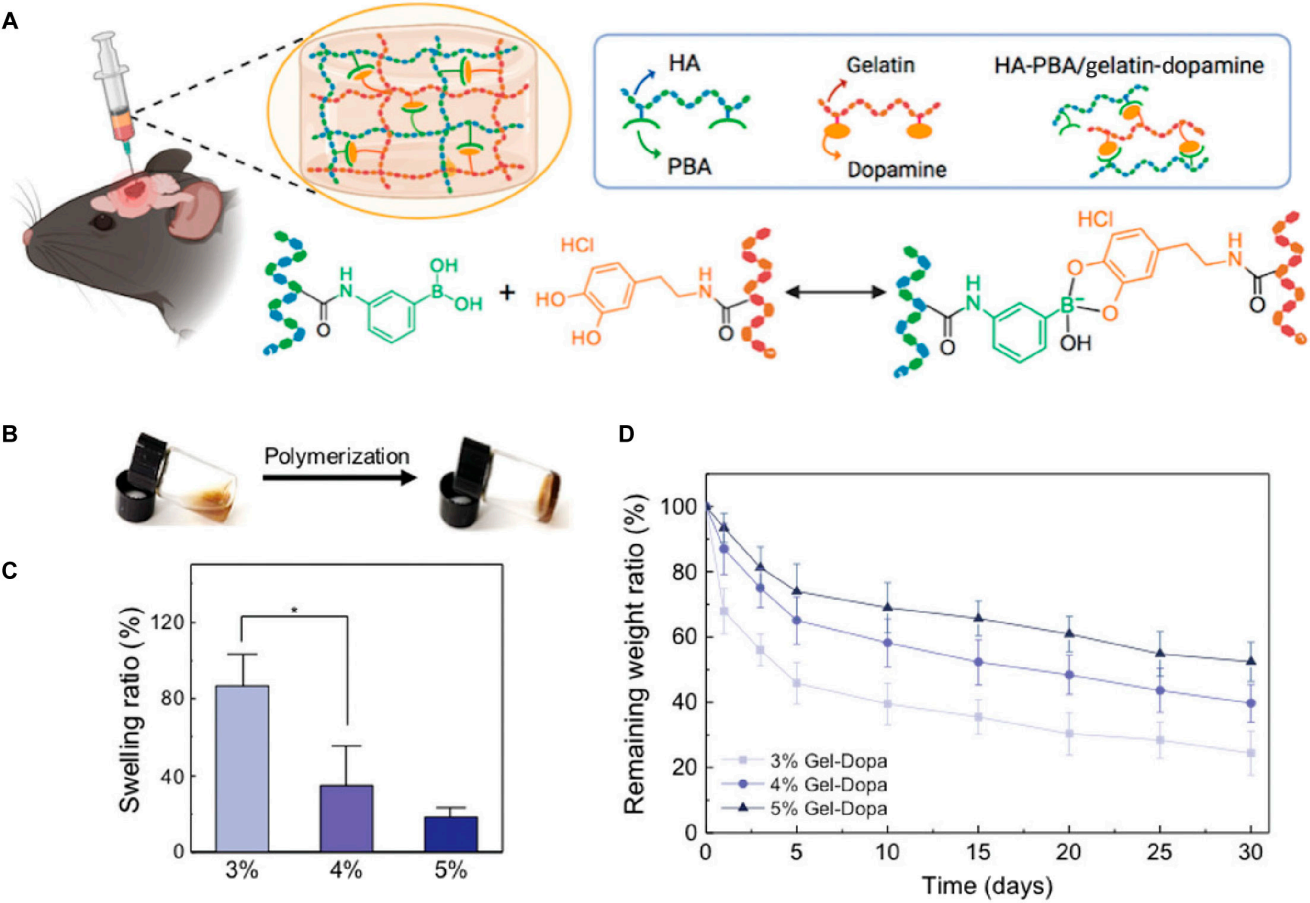
Characterization of HA-PBA/Gel-Dopa hydrogels. (A) Schematic of HA-PBA/Gel-Dopa hydrogel synthesis. (B) Gelation of HA-PBA/Gel-Dopa hydrogel. (C) Swelling ratios of fresh hydrogels (*n* = 5). (D) Degradation of hydrogels in PBS (*n* = 5). *P < 0.05, **P < 0.01, and ***P < 0.001, by one-way ANOVA [80]. Copyright 2022, Wiley.

The extracellular matrix (ECM) of the brain, synthesized by neurons and glial cells, establishes a perineuronal network by interweaving around neurons and incorporates crucial components such as hyaluronic acid (HA), chondroitin sulfate proteoglycan (CSPG), and connexins, crucial for maintaining the brain’s physiological functions [70–73]. Not only the ECM maintains tissue specificity but also, in recent years, biomimetic materials resembling the ECM have been developed for systemic repair applications [74–79]. Hu et al. [80] developed a hydrogel composed of dopamine (Dopa)-modified gelatin (Gel-Dopa) crosslinked with phenylboronic acid (PBA)-modified HA (HA-PBA), which closely mimics the composition of the brain ECM and shares similar mechanical properties, including hardness and viscoelasticity, with normal brain tissue (Fig. 2). In murine studies, this hydrogel showed effectiveness in promoting the healing of peri-traumatic tissues and facilitating nerve cell regeneration. Lainé et al. [24] formulated a HA-based hydrogel that reduces cortical tissue loss in the early stages of injury, slows lesion advancement, promotes significant vascularization by the host 1 week after implantation, enhances angiogenesis, diminishes neuroglial scar formation, and modulates astrocyte polarization to exert anti-inflammatory effects. Furthermore, Addington et al. [81] synthesized a HA-laminin (HA-LM) hydrogel. In vitro experimentation revealed heightened expression of the G protein-coupled receptor CXCR4 in neural progenitor/stem cells (NPSCs) within this hydrogel. This suggests an augmented NPSC response to stromal cell-derived factor-1α (SDF-1α) gradients, a chemokine known for its high expression in the brain and rapid concentration increase in cerebrospinal fluid after TBI [82,83]. Literature indicates that NPSCs are inclined to migrate toward SDF-1α [84]. Therefore, the HA-LM hydrogel substantiates its potential in NPSC transplantation studies for TBI treatment by enhancing NPSC migration in response to SDF-1α [81].

In addition to employing various materials to emulate the ECM, bioscaffolds can also be derived from decellularized animal tissues. ECM bioscaffolds, crafted from decellularized porcine spinal cord, optic nerve, and brain tissues, demonstrate cytocompatibility while preserving their functional integrity [85,86]. Ghuman et al. [87] utilized porcine bladder to fabricate ECM hydrogel. Animal studies established that this hydrogel persists at the site of a stroke lesion for an extended duration, curtails lesion expansion, and adheres to the lesion without impairing the behavioral functions of the subjects. Zhang et al. [88] procured urinary bladder matrix (UBM) from porcine bladder to generate an inductive hydrogel that mitigates lesion volume after TBI in rats, offering crucial structural support for the damaged brain. Furthermore, UBM hydrogel reduces white matter damage and enhances motor performance, although it does not significantly enhance cognitive functions in TBI-affected rats. Wu et al. [74] harvested brain-derived ECM from decellularized porcine brain tissue. In their study, implantation of this brain-derived ECM diminished lesion volume, preserved hippocampal CA3 neurons, lessened white matter damage, and ameliorated both motor and cognitive functions in mice. Moreover, it inhibits neural scar formation and diminishes neuroinflammation by modulating pro-inflammatory cellular responses.

Decellularized ECM hydrogels may also be utilized to produce infusible ECM (iECM) for intravascular injection. Spang et al. [89] employed decellularized myocardial ECM to fabricate ECM hydrogels; subsequently, low molecular weight iECM was derived through techniques such as centrifugation, dialysis, and aseptic filtration. Human blood compatibility assays confirmed that, akin to ECM hydrogels, iECM is compatible with blood [89,90]. In a TBI mouse model, iECM demonstrated the capability to localize specifically to the injury site and exhibited a brief blood half-life, facilitating elimination via hepatic and renal pathways. Importantly, in the context of TBI, disruption of the BBB may precipitate an influx of inflammatory cells and proteins into the brain, exacerbating neuroinflammation and cerebral edema [61,91]. Ex vivo experiments have indicated that iECM can diminish vascular permeability and foster neovascularization, thereby mitigating inflammatory responses [89].

### Stem cell-loaded hydrogels

Stem cells, which are undifferentiated cells present throughout all life stages, possess the capability to differentiate into a variety of tissues and organs. Based on their differentiation potential, these cells are categorized into several types: totipotent, pluripotent, multipotent, oligopotent, and unipotent [92]. Given their inherent abilities for self-renewal and differentiation, stem cells are frequently employed in the field of regenerative medicine research [93]. Mesenchymal stem cells (MSCs), which are readily obtainable from numerous tissues, are particularly favored in therapeutic research for TBI [94]. Wang et al. [95] treated HA with horseradish peroxidase (HRP) and galactosidase (GalOx) to prepare hyaluronic acid hydrogel (HT) and hyaluronic acid hydrogel with neurotrophic factor (NGF) (HT/NGF), and added bone marrow mesenchymal stem cells (BMSCs) into the hydrogel. It was verified that the HT hydrogel was biocompatible, and the addition of NGF significantly increased cell viability. The HT hydrogel loaded with BMSC and NGF promoted the recovery of neuromotor and learning memory functions in TBI mice, significantly reduced the neuroinflammation and apoptosis around the injury site, and strengthened the repair of the damaged tissues and neural regeneration, which might be related to the paracrine secretion of BMSC [95–98]. Further, Li et al. [99] developed a gelatin-based hydrogel using gelatin, HRP, and choline oxidase (ChOx), referred to as gelatin-hydroxybenzene (GH) hydrogel. In vitro investigations reveal that this hydrogel supports the neural differentiation of BMSCs and amplifies the expression of NGFs. When implanted in TBI models, GH hydrogel has demonstrated potential in enhancing motor and cognitive function recovery, reducing inflammation, elevating NGF levels, curbing apoptosis, stimulating neurogenesis, and improving the structural remodeling of afflicted regions [100].

**Fig. 3.**
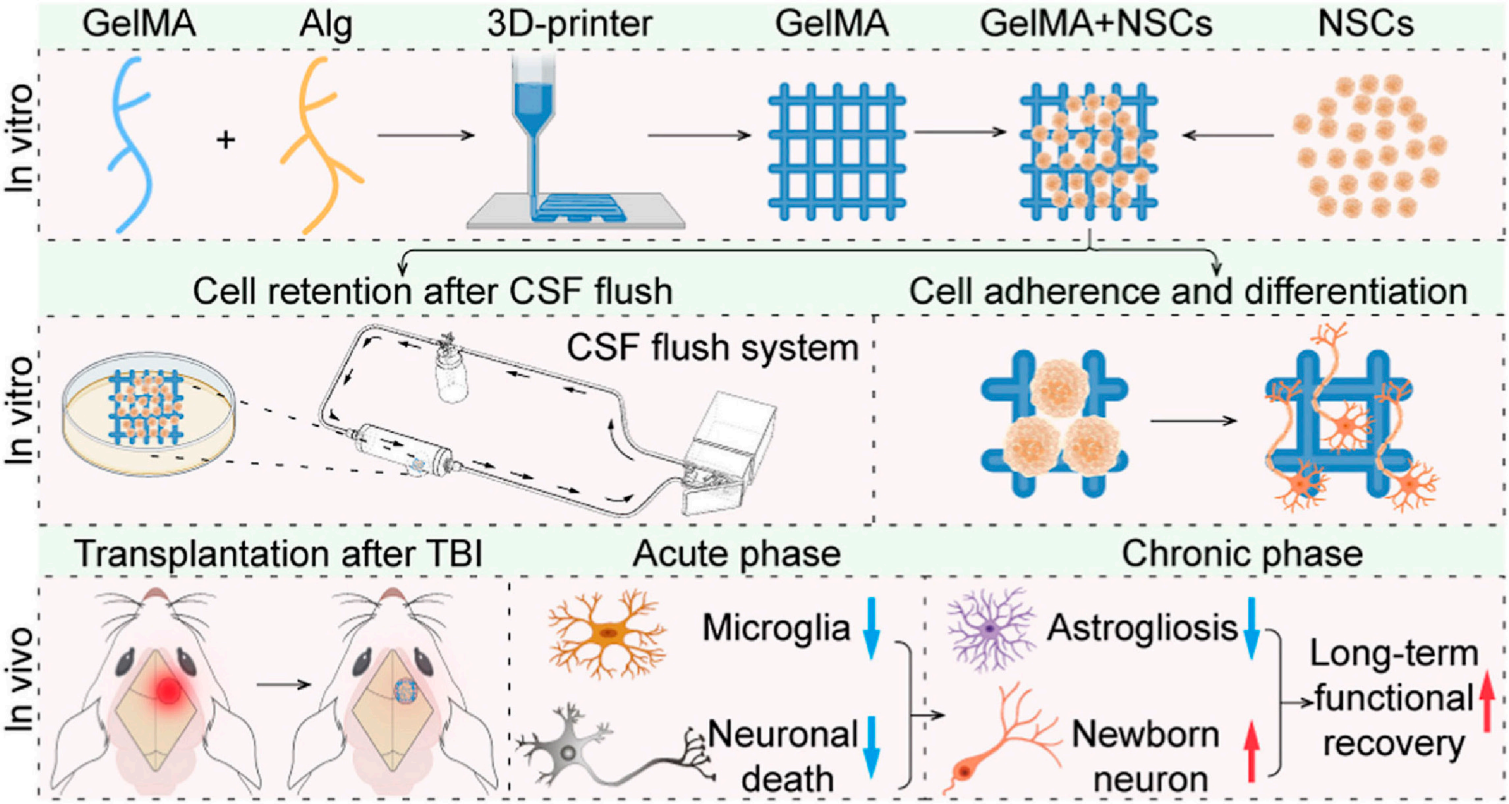
Loading NSCs on GelMA/Alg hydrogel and the curative effect of hydrogel on TBI SD rats [101]. Copyright 2023, Elsevier Ltd.

In addition to BMSCs, NSCs are frequently employed in research focusing on TBI treatment [67]. Chen et al. [101] isolated NSCs from neonatal mice, utilized 3-dimensional (3D) printing technology to fabricate 3 types of hydrogels, and subsequently cultured the NSCs on these hydrogel scaffolds. Their findings indicate that NSCs cultured on gelatin methacrylate (GelMA)/sodium alginate (Alg) hydrogel scaffolds exhibited superior adhesion, proliferation, and differentiation capabilities. Moreover, the GelMA/Alg hydrogel scaffold served as a neuroprotective agent by curtailing microglial activation and diminishing neuronal death during the acute phase of TBI. During the chronic phase, this hydrogel scaffold mitigated neuronal loss, reduced neuroglial scarring, and facilitated the generation of new neurons and vasculature (Fig. 3). Betancur et al. engineered a hydrogel using chondroitin sulfate glycosaminoglycans (CS-GAG) derived from brain ECM for experimental purposes. The comparative study revealed that, 4 weeks after TBI, rats implanted with the CS-GAG matrix displayed reduced neural tissue damage and a higher survival and proliferation rate of NSCs, compared to rats with a nonimplanted CS-GAG matrix. Furthermore, these implanted rats exhibited an attenuated inflammatory response, reduced astrocyte scarring, and greater retention of local fibroblast growth factor-2 (FGF-2), which plays a critical role in the mitosis and differentiation of NSCs into neurons and glial cells [102,103].

Exosomes are extracellular vesicles secreted by a diverse array of cells [104]. Research into the utility of stem cell-derived exosomes for TBI therapy is ongoing [105,106]. Liu and colleagues [107] synthesized DHA-Col (DHC) hydrogels by chemically modifying HA with aldehyde and methacrylate groups. BMSCs were isolated and cultured from Sprague-Dawley rats aged 5 to 7 d, and exosomes were subsequently extracted from these cells via continuous centrifugation [108,109]. In vitro experiments demonstrated that NSCs not only assimilate these BMSC-derived exosomes (BME) but also preferentially differentiate into neurons and oligodendrocytes under their influence, while the differentiation into astrocytes is inhibited. In a TBI rat model, the DHC hydrogel was shown to enhance NSC recruitment and neuronal regeneration at the injury sites. The addition of BME to the hydrogel significantly amplified these effects and led to a marked increase in vascular endothelial growth factor (VEGF), indicative of enhanced angiogenesis [110]. Moreover, the inhibitory effect of BME on astrocyte differentiation reduces the formation of glial scars, thereby facilitating structural remodeling of the brain [111].

### Drug-loaded hydrogels

**Fig. 4.**
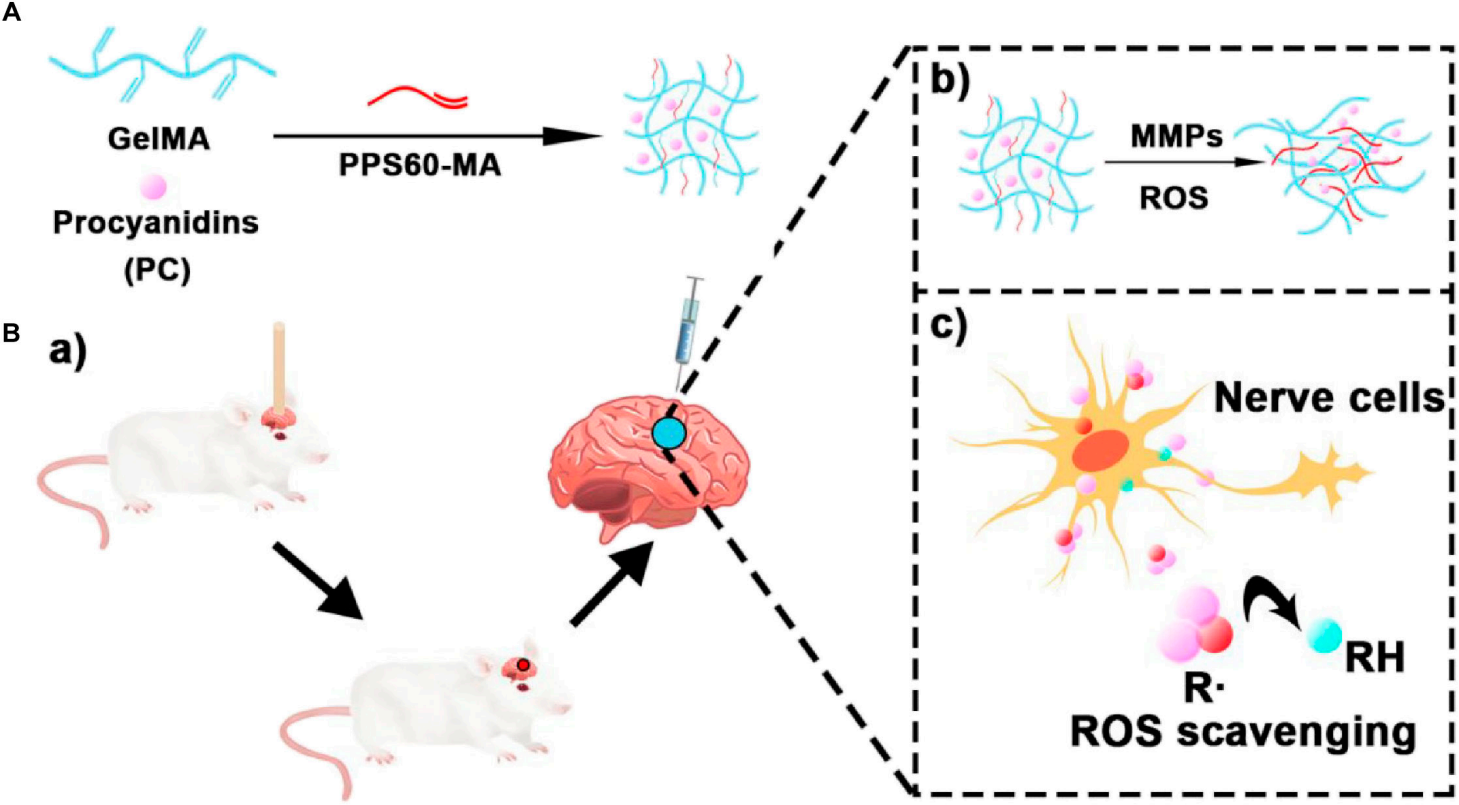
(A) Schematic diagram of GelMA-PPS/PC hydrogel preparation process by blue light cross-linking. (B) Schematic illustration of establishing a TBI model. (a) Schematic diagram of the TBI model and injection of GelMA-PPS/PC hydrogel in the damaged area. (b) In the microenvironment of brain injury, the GelMA-PPS/PC hydrogel releases PCs under matrix metalloproteinase (MMP) and ROS conditions, (c) synergistically depletes ROS, and enhances the protective effect of nerve cells after brain injury [119]. Copyright 2022, American Chemical Society.

Compared to conventional drug delivery methods, hydrogel-based drug delivery systems offer precise targeting to diseased tissues, enabling localized high drug concentrations while minimizing damage to healthy tissues [112]. Hydrogels loaded with pharmaceutical agents have also been investigated in the context of TBI treatment. Following TBI, a rapid escalation in glutamate levels induces excitotoxicity, excessive production of ROS, and mitochondrial dysfunction, which collectively contribute to secondary brain damage [113–118]. Huang et al. [119] developed a GelMA-poly(propylene sulfone) (PPS) hydrogel containing procyanidin (PC) (Fig. 4A). In vitro studies showed that the GelMA-PPS/PC hydrogel effectively scavenged ROS. Moreover, in vivo applications of this hydrogel significantly enhanced the antioxidant capacity of brain tissues and ameliorated tissue damage attributed to ROS (Fig. 4B). The subsequent reduction in ROS levels mitigated the damage to the BBB caused by TBI, thereby diminishing cerebral edema and the inflammatory response [120,121]. Qiu et al. [25] created a HA-PBA/PVA hydrogel by coupling 3-aminobenzeneboronic acid (PBA) with HA and integrating polyvinyl alcohol (PVA). This formulation was used to encapsulate desferrioxamine methanesulfonate (DFO), which reacts with ROS, reverses iron deposition, reduces the depletion of superoxide dismutase (SOD) and glutathione (GSH), and lowers ROS levels. This intervention prevents brain iron overload and reduces oxidative stress.

GCs have long been utilized in the management of TBI, yet their application remains contentious due to potential adverse prognostic outcomes and systemic side effects associated with high dosages of GCs [122–124]. Research has indicated that low doses of dexamethasone (DXM) may be employed effectively in TBI treatment [125]. In a notable study, Jeong et al. [126] incorporated DXM into HA, subsequently crosslinking it with poly(ethylene glycol) (PEG)-bis-(acryloyloxy acetate) (AA) to create a PEG-bis-AA/HA-DXM hydrogel. This hydrogel demonstrated a reduction in the levels of pro-inflammatory cytokines such as IL-1β, transforming growth factor (TGF-β), and TNF-α, indicating its potential to suppress inflammatory mediator secretion. Additionally, the same study found that the PEG-bis-AA/HA-DXM hydrogel significantly up-regulated the expression of the anti-apoptotic protein BCL2, thereby inhibiting apoptosis.

Hydrogen sulfide (H_2_S), an endogenous signaling molecule, has been recognized for its ability to activate antioxidant enzymes and curtail free radical reactions, thus diminishing oxidative stress. Furthermore, it offers neuroprotection by modulating apoptosis-related genes to decrease apoptosis [127]. Experimental evidence suggests that H_2_S exhibits a therapeutic effect in TBI-afflicted mice [128]. In their research, Chen et al. [129] developed an H_2_S/silk fibroin (SF) hydrogel by extracting filipin protein from raw silk fibers. This hydrogel was shown to suppress the expression of H_2_S synthase in neurons affected by TBI, mitigate neurodegenerative lesions and cerebral edema, enhance motor and cognitive functions, and diminish tissue damage and neuroinflammation after TBI.

### Self-assembled peptide hydrogels

**Fig. 5.**
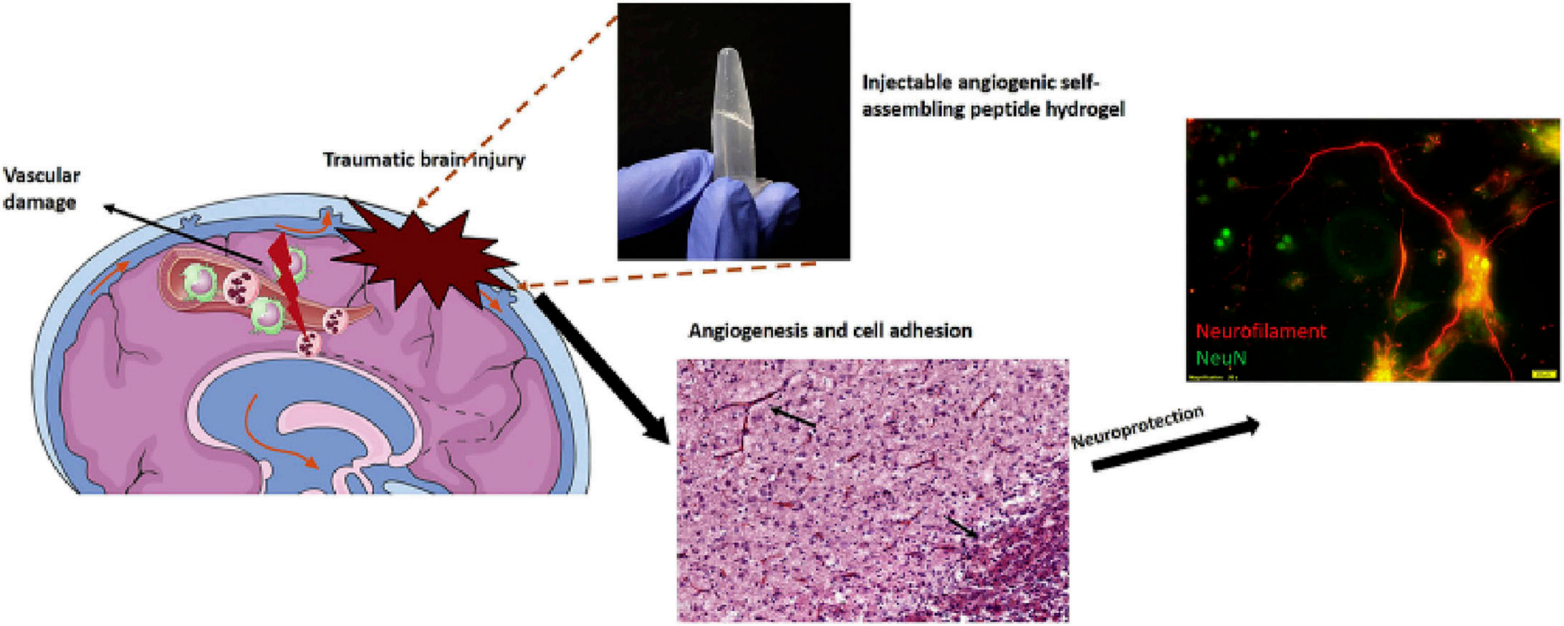
Angiogenic peptide hydrogels for treatment of TBI [133]. Copyright 2020, KeAi Communications Co. Ltd.

Self-assembled peptide hydrogels (SAPHs) constitute a class of supramolecular hydrogels that facilitate neovascularization and can incorporate cells, ECM, and growth factors [130–134]. Ma et al. developed SLanc hydrogels that transition to an in situ hydrogel immediately upon injection and maintain their integrity for up to 14 d. The incorporation of angiogenic peptides into these hydrogels significantly up-regulates vascular endothelial growth factor receptor-2 (VEGFR2) and increases the number of endothelial cells. This, in turn, promotes angiogenesis and protects nerves (Fig. 5) [133–135]. These effects align with the known angiogenesis-enhancing properties of VEGFR2 [136,137]. In a related study, Jahanbazi Jahan-Abad et al. [138] harvested human neural stem/progenitor cells (hNS/PCs) from the proximal mesial temporal lobe tissues of patients with refractory temporal lobe epilepsy. These cells were encapsulated in PuraMatrix peptide (PM) hydrogels, demonstrating enhanced survival rates of the implanted cells. Moreover, the PM hydrogel, when used in conjunction with hNS/PC, decreased the volume of brain lesions and significantly reduced levels of neuroinflammatory markers, including TNF-α, IL-1α, IL-6, and glial fibrillary acidic protein (GFAP), thereby aiding in the suppression of neuroinflammation. Sarkar et al. [139] engineered a neuroprotective peptide (SLen), which, upon implantation in rats with TBI, promoted neuronal growth and mitigated cortical atrophy, underscoring the therapeutic potential of peptide hydrogels in neuroprotection and brain recovery processes.

### Conductive hydrogels

**Fig. 6.**
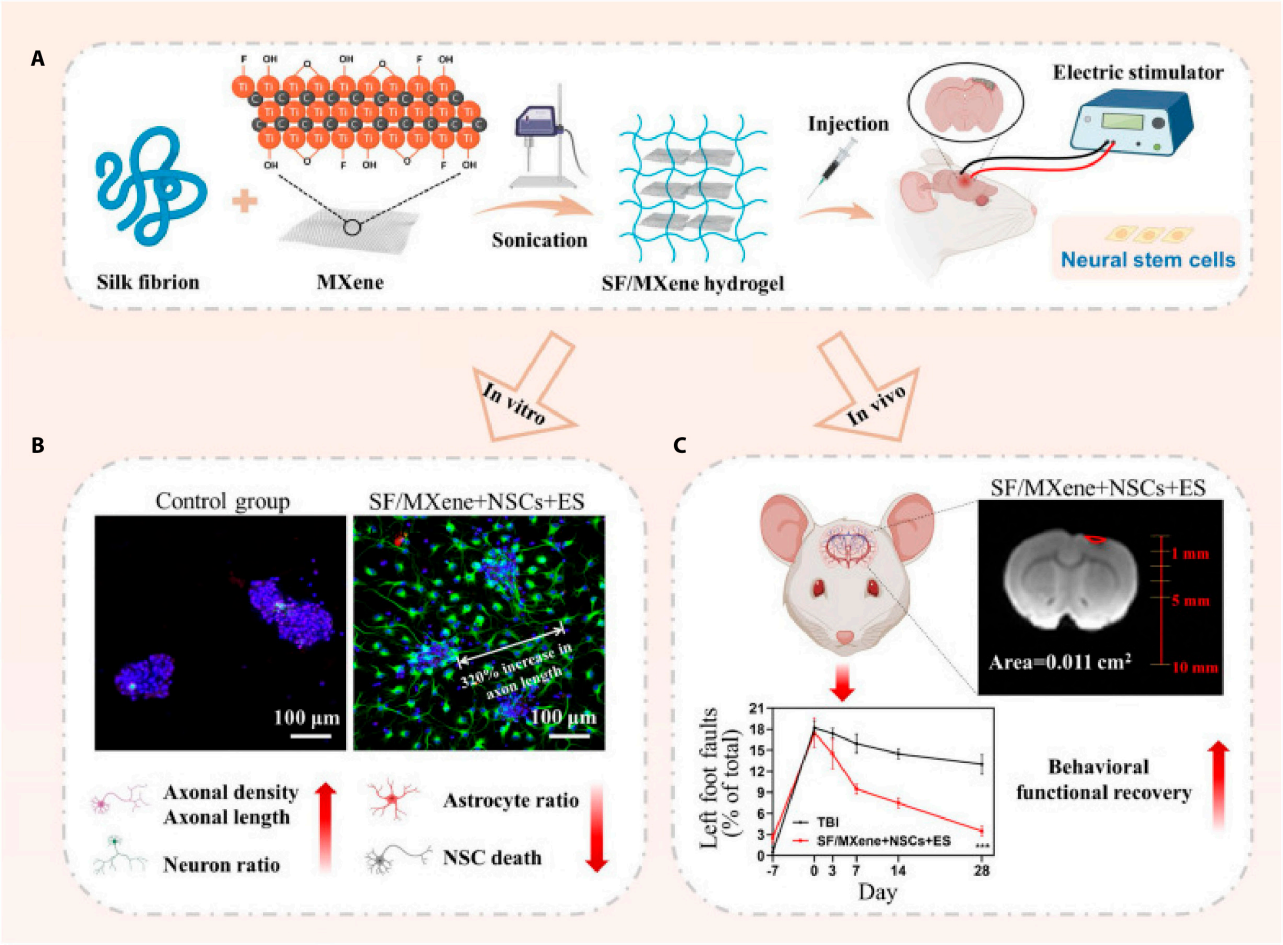
Schematic illustration. (A) Preparation process of SF/MXene hydrogel. (B) Images of NSC neural differentiation on SF/MXene hydrogel with ES in vitro. (C) SF/MXene hydrogel combined NSCs with ES to treat TBI [143]. Copyright 2024, BioMed Central.

Stimuli-responsive hydrogels represent a category of advanced hydrogels capable of undergoing stimulus-induced alterations in volume and structure in response to various external factors, including temperature, electric fields, magnetic fields, pH, light, mechanical forces, or pathological changes. These hydrogels are extensively utilized in diverse biomedical research applications [140,141]. Notably, conductive hydrogels constitute a subset of stimuli-responsive hydrogels characterized by their exceptional electrical conductivity. Tanikawa et al. [142] synthesized a hydrogel composed of cationic and anionic monomers in a 1:1 ratio (referred to as C1A1 hydrogel), which was subsequently frozen to generate a porous structure that achieved conductivity comparable to that of pure water. It was observed that the positive and negative charges within the C1A1 hydrogel were nearly balanced. In animal studies, this hydrogel was directly implanted into a TBI mouse model, serving as a scaffold to mitigate further tissue loss. When the hydrogel was infused with VEGF and subsequently implanted into the mouse brain, significant neovascularization was observed; however, the formation of neurons and glial cells was not evident. Conversely, when C1A1 hydrogels were loaded with NSCs, pronounced neuronal differentiation and migration were clearly documented. Yang et al. [143] synthesized an electrically conductive hydrogel, designated SF/MXene, incorporating filipin protein and MXene (Ti3C2Tx) (Fig. 6A). This SF/MXene hydrogel, when loaded with NSCs and subjected to electrical stimulation (ES), demonstrated in vitro that ES significantly facilitated the differentiation of NSCs into neurons while inhibiting their differentiation into astrocytes (Fig. 6B). In animal experiments, the treated lesions exhibited a marked reduction in volume, the integrity of the BBB was preserved, neuromotor functions were effectively restored, and optimal outcomes were achieved through the combined treatment approach (Fig. 6C).

## Conclusions and Prospects

Currently, there is a notable deficiency in treatments with clear efficacy for TBI, underscoring the urgent need to identify and develop effective therapeutic strategies. This review succinctly presents the primary pathophysiological changes associated with TBI while focusing on recent advancements in the utilization of hydrogels in TBI therapy. As an innovative biomaterial, hydrogels have gained considerable interest in the field of regenerative medicine. Initially, the therapeutic potential of ECM hydrogels, which are devoid of additional components and leverage the intrinsic bioactivity of the ECM, is discussed in the context of ameliorating TBI in animal models. Subsequently, a comprehensive review of stem cell-loaded hydrogels, drug-loaded hydrogels, SAPHs, and conductive hydrogels elucidated their pivotal role in mitigating tissue damage, alleviating inflammation and oxidative stress, inhibiting apoptosis, and promoting neurogenesis and angiogenesis (Table). Furthermore, hydrogels exhibit significant advantages as scaffolds for the treatment of brain tissue injuries, including enhanced retention of stem cells during transplantation therapies and improved metabolic stability and bioavailability of pharmaceuticals when employed as drug carriers. The application of hydrogels for pharmacological treatment of TBI can mitigate the challenge of drug delivery across the BBB, enhance local drug concentration at the injury site, minimize enzymatic degradation of drugs within the body, and diminish the systemic toxicity associated with conventional medication. Additionally, hydrogels can regulate the release of pharmaceuticals through their 3D mesh architecture, thereby facilitating sustained-release effects and extending the duration of drug action within the body. In addition to hydrogels, nanoparticles represent a prominent category of drug carriers that are also employed in therapeutic investigations for TBI. Existing research indicates that nanoparticles exhibit comparable advantages to hydrogels in terms of targeted drug delivery to the brain, reduction of drug dosage, and enhancement of bioavailability. The distinctions between hydrogels and nanoparticles as drug carriers for TBI applications are as follows: First, hydrogels are directly administered to the affected regions of brain tissue via injections or other methods, whereas nanoparticles can be directed to the brain through the BBB. However, the capacity of nanoparticles to accumulate in the damaged brain tissue across the BBB is influenced by factors such as material composition, volume, and other characteristics [144]. Furthermore, hydrogels are more effective than nanomaterials in controlling the sustained release of drugs; the release duration of drugs encapsulated within hydrogels can extend up to 1 week or longer, whereas the retention time of nanoparticle-delivered drugs in the brain typically spans hours or days [119,126,145,146]. Most critically, hydrogels demonstrate superior biocompatibility and biodegradability, while certain nanoparticles may exhibit toxicity and potential accumulation in the brain, rendering them less safe compared to biodegradable hydrogels [147–149]. In light of these attributes, hydrogels are deemed more appropriate for the treatment of TBI.

**Table.** Therapeutic mechanism of hydrogel for traumatic brain injury

Categories	Hydrogels	Models	Outcome	References
ECM-based hydrogel	HA-PBA/Gel-Dopa	C57BL/6J mice	• Promoted tissue healing	[80]
			• Promoted neurogenesis	
	HA	C57BL/6 mice	• Reduced tissue damage	[24]
			• Inhibited the formation of glial scars	
			• Promoted angiogenesis	
	HA-LM	In vitro	• Improved the response of NPSC to SDF-1α	[81]
	UBM-ECM	SD rats	• Reduced tissue damage	[87]
			• Inhibited the formation of glial scars	
			• Improved motor function	
			• Anti-neuroinflammation	
	UBM	SD rats	• Reduced tissue damage	[88]
			• Reduced white matter damage	
			• Improved motor function	
	Brain-derived ECM	C57BL/6J mice	• Reduced tissue damage	[74]
			• Reduced white matter damage	
			• Improved motor and cognitive function	
			• Inhibited the formation of glial scars	
			• Anti-neuroinflammation	
	iECM	In vitro	• Decreased vascular permeability	[89]
			• Promoted angiogenesis	
			• Anti-neuroinflammation	
Stem cell-loaded hydrogel	HT/NGF/BMSC	C57BL/6 mice	• Improved motor and cognitive function	[95]
			• Anti-neuroinflammation	
			• Anti-apoptosis	
			• Promoted neurogenesis	
			• Promoted tissue healing	
	GH/BMSC	In vitro and C57BL/6 mice	• Improved motor and cognitive function	[100]
			• Anti-neuroinflammation	
			• Anti-apoptosis	
			• Improved the secretion of neurofactors	
			• Promoted neurogenesis	
			• Improved structural remodeling	
	GelMA/Alg/NSC	SD rats	• Reduced tissue damage	[101]
			• Reduced neuronal death	
			• Inhibited the activation of microglia	
			• Promoted angiogenesis	
			• Promoted neurogenesis	
	CS-GAG/NSC	SD rats	• Promoted the survival and proliferation of NSC	[102]
			• Reduced tissue damage	
			• Improved local FGF2 retention	
			• Anti-neuroinflammation	
			• Inhibited the formation of glial scars	
	DHC-BME	SD rats	• Enhanced recruitment of endogenous NSCS	[110]
			• Inhibited the formation of glial scars	
			• Promoted angiogenesis	
			• Promoted neurogenesis	
			• Improved structural remodeling	
Drug-loaded hydrogel	GelMA-PPS/PC	ICR mice	• Anti-oxidative stress	[119]
			• Anti-neuroinflammation	
			• Protected the BBB	
	HA-PBA/PVA/DFO	SD rats	• Anti-oxidative stress	[25]
			• Inhibited iron overload	
	PEG-bis-AA/HA-DXM	SD rats	• Reduced tissue damage	[126]
			• Improved motor function	
			• Anti-neuroinflammation	
			• Anti-apoptosis	
	H_2_S/SF	ICR mice	• Reduced tissue damage	[129]
			• Improved motor and cognitive function	
			• Anti-neuroinflammation	
Self-assembled peptide hydrogel	Slanc	SD rats	• Promoted angiogenesis	[133]
			• Protected nerve	
	PM-hNS/PC	Wistar rats	• Promoted the survival of transplanted cells	[138]
			• Reduced tissue damage	
			• Anti-neuroinflammation	
	Slen	SD rats	• Prevented cortical atrophy	[139]
			• Promoted neurogenesis	
Conductive hydrogels	C1A1/VEGF/NSCs	In vitro C57BL/6J mice NOD mice	• Reduced tissue damage	[142]
			• Promoted angiogenesis	
			• Promoted neuronal differentiation	
	SF/MXene/NSCs/ES	In vitro and SD rats	• Promoted neuronal differentiation	[143]
			• Reduced tissue damage	
			• Protected the BBB	

Hydrogels have shown considerable promise in preclinical studies of TBI; however, several challenges need to be addressed to effectively translate these findings into clinical applications for TBI patients. First, existing research on hydrogels for the treatment of TBI has predominantly focused on in vitro experiments to assess hydrogel degradation and drug metabolism, with a notable absence of in vivo monitoring. This gap could potentially be addressed in numerous other studies. Park et al. [150] have pioneered a dual-channel fluorescence imaging technique that employs a near-infrared brain-specific contrast agent integrated with a hydrogel, facilitating concurrent observation of hydrogel degradation and the ingrowth of brain tissue. Furthermore, various studies have demonstrated that hydrogel formation, degradation, and drug release in vivo can be effectively monitored through the utilization of fluorescent or other contrast agents [151–154]. The integration of therapeutic interventions with monitoring capabilities represents a promising avenue for future research in the application of hydrogels in TBI. Second, the physical properties of hydrogels do not align precisely with those of brain tissue; therefore, when utilized for TBI, characteristics such as hardness and adhesion warrant further investigation. Finally, while the biocompatibility of hydrogels has been established in current animal experiments, their long-term safety in patients with TBI remains uncertain, and the potential toxicity of hydrogel degradation products necessitates further investigation.

Although the utilization of hydrogels for the treatment of TBI remains in the preclinical phase, their considerable potential has become increasingly evident. Owing to its remarkable biocompatibility, hydrogels play a pivotal role in providing structural support to the injured brain, mitigating the loss of compromised tissues, while concurrently serving as a vehicle to augment the efficacy of pharmacological agents or stem cells in the context of TBI. Half of these investigations have employed hydrogels in conjunction with stem cell therapy, demonstrating significant efficacy in modulating neuroinflammation and facilitating neurogenesis. Future research endeavors should prioritize enhancing the biosafety and biocompatibility of hydrogels to facilitate their expedited translation into clinical practice as a viable treatment modality for TBI.
